# Longitudinally extensive transverse myelitis as a manifestation of neuro-chikungunya in a lupus patient: a case report

**DOI:** 10.3389/fmed.2025.1600806

**Published:** 2025-08-18

**Authors:** Emanuelle de Matos Rodrigues, Lysiane Maria Adeodato Ramos Fontenelle, Andrea Rocha de Saboia MontAlverne, Kirla Wagner Poti Gomes, Samia Araújo de Sousa Studart, Beatriz Rodrigues Neri, Carlos Ewerton Maia Rodrigues

**Affiliations:** ^1^Resident in Rheumatology, Rheumatology Division, Hospital Geral de Fortaleza, Fortaleza, Brazil; ^2^Graduate Program in Medical Sciences, Universidade de Fortaleza (Unifor), Fortaleza, Brazil and Rheumatology Division, Hospital Geral de Fortaleza, Fortaleza, Brazil; ^3^Rheumatology Division, Hospital Geral de Fortaleza, Fortaleza, Brazil; ^4^Medical School, Universidade de Fortaleza, Fortaleza, Brazil; ^5^Graduate Program in Medical Sciences, Universidade de Fortaleza (UNIFOR), Fortaleza, Brazil

**Keywords:** systemic lupus erythematosus, chikungunya virus, transverse myelitis, myeloneuropathy, outcome

## Abstract

**Background:**

Myeloneuropathy is rare complication of Chikungunya virus (CHIKV) infection which may have an underlying immune-mediated pathogenesis.

**Case presentation:**

We describe a 31-year old female patient with systemic lupus erythematosus (SLE) presenting with acute/hyperacute bulbar and medullary fever syndrome with ascending tetraparesis associated with arthritis, requiring differential diagnosis. The analysis of the cerebrospinal fluid (CSF) revealed inflammatory markers, high protein levels, high lymphocyte and neutrophil counts, and reduced glucose. Neuroimaging showed amorphous foci of hyperintensity in T2, suggesting extensive medullary edema, especially in the central region, with no significant contrast enhancement, along with areas of medullary atrophy consistent with holocord longitudinally extensive transverse myelitis and periventricular and periaqueductal involvement. CHIKV IgM antibodies were also detected in the CSF.

**Conclusion:**

The present case highlights the importance of investigating atypical neurological syndromes in SLE and of including CHIKV infection, though rare, in the differential diagnosis of patients from endemic regions in order to minimize morbidity and mortality.

## Background

Systemic lupus erythematosus (SLE) is an autoimmune disease compromising multiple systems, including the central nervous system and the peripheral nervous system (1, 2). Myelopathy, a rare neurological condition affecting 1–2% of SLE patients (3, 4), is referred to as ‘transverse' when the inflammation extends horizontally throughout the cross-section of the spinal cord, and ‘longitudinally extensive transverse myelitis' (LETM) when it extends over three or more vertebral segments. LETM is considered secondary to SLE when associated with inflammatory markers detected in the cerebrospinal fluid (CSF), pleocytosis, and raised IgG levels on gadolinium-enhanced nuclear MRI, provided other autoimmune and infectious causes have been ruled out (4). The physiopathology of the inflammatory process remains unclear, but some authors believe that both vascular and inflammatory components play a role (3).

The chikungunya virus (CHIKV) is a togaviridae RNA virus of the genus *Alphavirus* transmitted by the mosquito species *Aedes aegypti*. The term ‘neuro-chikungunya' covers a set of neurological clinical syndromes observed in up to 12% of carriers (5). Predominant manifestations include encephalitis, Guillain-Barré syndrome, myelitis, meningoencephalitis and optic neuropathy (6–10). Clinical suspicion may be confirmed by anti-CHIKV IgM positivity or RT-PCR of the CSF. On MRI, patients with CHIKV-induced myelopathy usually display hyperintense lesions in T2, with peripheral enhancement and perivascular distribution in the spinal cord (7). A meta-analysis of 19 studies (7,319 patients) on CHIKV in neurological disorders found that the frequency of CHIKV in neurological clinical subgroups was higher among patients with myelitis (27%), acute disseminated encephalomyelitis (27%) and Guillain-Barré syndrome (15%) (5). Importantly, no case has been published of the association of CHIKV-associated myelopathy in patients with SLE.

In this report we present the clinical findings of a case of neuroinvasive CHIKV infection in an SLE patient, highlight the importance of differentiating the expression of SLE flares from neuropsychiatric symptoms secondary to CHIKV, and explore the peculiarities of the latter. CHIKV should be considered in the differential diagnosis of myelopathy in endemic areas. Considering the inexistence of published cases, the present report is intended as a contribution to the medical debate on the diagnosis and treatment of neuro-chikungunya in this patient population.

## Case presentation

A 31-year-old woman from Fortaleza (Northeastern Brazil) diagnosed since 2009 with SLE and meeting the 2019 ACR/EULAR criteria for SLE (11) (arthritis, serositis, oral ulcers, alopecia, fever, hemolytic anemia associated low complement c3 and c4, and anti-DNA positivity), without adequate disease monitoring, presented at an emergency service with asthenia, anorexia, weight loss, progressive dyspnea and lipothymia, reportedly starting 4 months earlier, and was referred to a local tertiary-level facility. At the time of admission, the patient was using prednisone (10 mg/day) and had been using chloroquine diphosphate (250 mg/day) for 2 years.

During hospitalization, the patient started experiencing intermittent fever (38–39.5°C), without chills or rigor, but responsive do antipyretics. Nausea and vomiting were present, but not hiccups. Seven days after fever onset, arthritis worsened acutely, accompanied by tremors, painful spasms, allodynia, dysesthesia, and lower limb weakness, followed by dysphagia, central nystagmus and urinary retention. Within 24 h, the neurological condition deteriorated, with progression to respiratory distress and risk of acute respiratory failure, requiring orotracheal intubation and transfer to the ICU.

Upon neurological examination, the patient had multidirectional (mostly horizontal) nystagmus and absent gag reflex on the right side. The motor examination revealed no abnormal movements, grade 2 muscle strength, and intense joint pain. The reflexes were preserved, with no dysmetria or signs of meningeal irritation, scoring 5 on the modified Rankin scale (mRS).

Due to acute/hyperacute bulbar and medullary fever syndrome and ascending tetraparesis associated with arthritis, the patient was submitted to a neuraxis MRI and CSF analysis of cellularity, infectious agents and antibody titers. In addition, an ophthalmological examination was performed. Care was provided by a multidisciplinary team including a neurologist.

**Figure 1:**
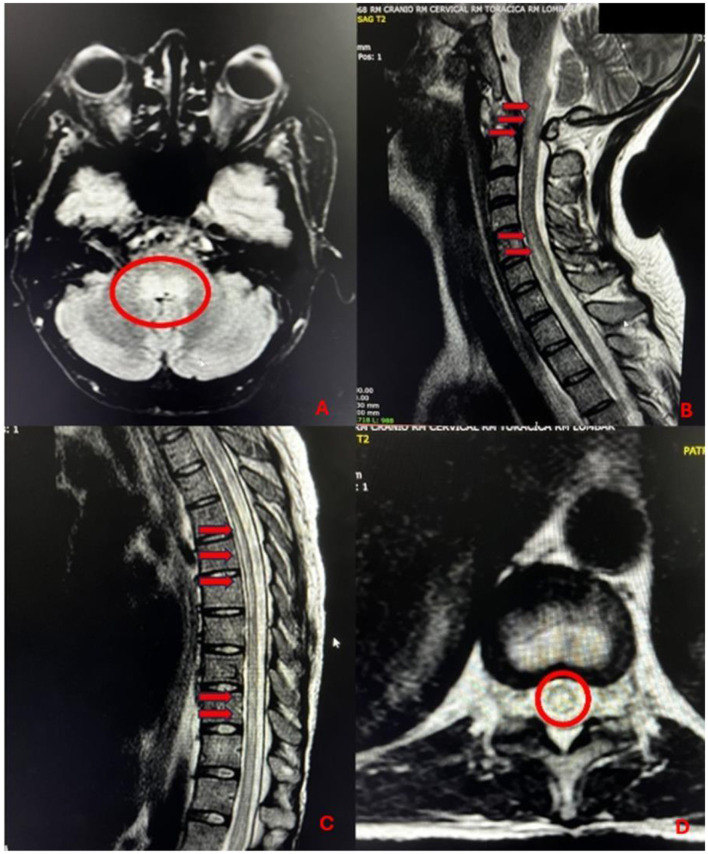
**(A)** T2 and FLAIR hyperintensity in the dorsal region of the pons, medulla oblongata and periependymal region of the fourth ventricle (red circles). **(B)** Isolated hyperintensive amorphous foci on FLAIR in the right semi-oval center, discontinuous signal changes in the central spinal cord, characterized by T2 hyperintensity from the pons to C2, and from C4 to C7, with slightly increased cord caliber in the latter. **(C, D)** Lower thoracic cord and conus medullaris displaying areas of signal change, characterized by T2 hyperintensity (red arrows).

On neuroimaging, the spinal cord displayed normal caliber and amorphous hyperintense foci in T2, suggesting extensive medullary edema, especially in the central region, with no significant contrast enhancement (Figure 1). These findings are consistent with holocord LETM and periventricular and periaqueductal involvement, along with areas of medullary atrophy suggestive of viral infection, possibly arbovirus. The CSF analysis revealed 258 nucleated cells, with a differential count of 48% neutrophils, 49% lymphocytes, and 3% monocytes. Adenosine deaminase (ADA) was 2.8 U/L, the glucose concentration was 29 mg/dL, and the total protein level was 371 mg/dL. The opening pressure was 25 cmH_2_O, and bacterioscopy and bacterial and mycobacterial culture were negative. Multiplex^®^ PCR was performed to screen for pathogens commonly associated with infectious meningoencephalitis. The panel included 14 agents (*Streptococcus pneumoniae, Streptococcus agalactiae, Escherichia coli K1, Haemophilus influenzae, Listeria monocytogenes*, cytomegalovirus, enterovirus, herpes simplex virus types 1 and 2, human herpesvirus 6, human parechovirus, varicella-zoster virus, and C*ryptococcus neoformans/gattii*). GeneXpert^®^ PCR was used for the detection of *Mycobacterium tuberculosis*. All tests were negative. Antibodies to CHIKV IgM were detected in the CSF but was negative for Dengue virus and Zika virus. RT-PCR for chikungunya, Zika virus and Dengue virus were negative in the CSF. Serology was non-conclusive for chikungunya (IgG and IgM) and negative for Dengue virus and Zika virus. Optic neuritis was ruled out on MRI and by the absence of aquaporin 4 antibodies (Table 1). In view of the subsequent appearance of signs of lupus nephritis (24-h proteinuria of 1,760 mg, complement consumption, reduced urine output), the patient was given pulse therapy with methylprednisolone at 1,000 mg per day for 5 days, plasmapheresis and cyclophosphamide at 1,000 mg (6 cycles). After 20 days of treatment, the patient was successfully extubated, followed by partial recovery of upper limb strength, despite the persistence of paraparesis (mRS = 4). At the time of writing, 18 months after the initial event, the neurological sequelae persist. The patient has progressed from tetraplegia to lower limb paraplegia but still requires a wheelchair for mobility and intermittent bladder catheterization due to a neurogenic bladder. Currently, improvement is observed in upper limb muscle strength, with strength graded as IV.

**Table 1:** Clinical and laboratory findings, treatment, and outcome.

**Author**	**Patient profile**	**Clinical characteristics**	**Laboratory features**	**Neuroimaging**	**Treatment**	**Clinical outcome**
Rodrigues et al. (2025)	Female; 31 years; 2009 diagnosis of cutaneous articular SLE	Fever (38 °C−39.5 °C), nausea and vomiting. 7 days later arthritis worsened acutely, accompanied by tremors, painful spasms, allodynia, dysesthesia, and lower limb weakness, followed by dysphagia, central nystagmus and urinary retention. Acute/hyperacute bulbar and medullary fever syndrome.	The CSF revealed high levels of protein, lymphocytes and neutrophils, and reduced glucose. Bacterioscopy, bacterial and mycobacterial culture, and Multiplex^®^ PCR and GeneXpert^®^ PCR were negative. CHIKV IgM antibodies positive in CSF, negative for Dengue virus and Zika virus. Aquaporin 4 antibodies negative in serum.	The spinal cord displayed normal caliber and amorphous hyperintense foci in T2, suggesting extensive medullary edema, especially in the central region, with no significant contrast enhancement.	Pulse therapy w/MP 1,000 mg for 5 days; 6 cycles of CP 1,000 mg followed by MMF 2 g/day.	Partial improvement mRS = 4

SLE, systemic lupus erythematosus; CSF, cerebrospinal fluid; CHIKV, chikungunya virus; MP, methylprednisolone; CP, cyclophosphamide; MMF, mycophenolate mofetil; mRS, modified Ranking scale.

## Discussion

In this report we describe an SLE patient developing LETM as the likely result of CHIKV infection. The condition was severe and disabling, with high risk of morbidity and mortality, and required aggressive treatment and careful etiological investigation, which revealed positivity for CHIKV IgM antibodies in CSF.

Neurological manifestations are rare (up to 2%) in SLE (4). In this subgroup, the neurological symptoms are the earliest manifestation of the disease in up to 30% of cases, or they may appear 1 or 2 years after diagnosis (2, 4). In contrast, our patient had been diagnosed with SLE 14 years earlier and had no history of SLE-related neurological or psychiatric symptoms.

Epidemiological studies show that myelopathy in SLE patients affects predominantly women in the third decade of life (4). On the other hand, myelopathy secondary to CHIKV infection affects mostly men, also in the third decade of life (mean ~26.3 years) (5).

Whether secondary to SLE or CHIKV, the signs and symptoms of LETM tend to be similar: essentially acute weakness of the limbs (especially the legs) associated with heightened sensitivity and sphincter changes (1, 3, 12). However, Chandak et al. (12) and Mehta et al. (13) observed that LETM secondary to CHIKV is usually preceded by systemic fever, joint pain and sometimes skin rash about 20 days to 3 weeks earlier.

The CSF analysis performed during inflammatory LETM revealed polymorphic cell pleocytosis associated with hypoglycorrhachia and increased protein levels (3, 13, 14). Such unspecific findings do not in themselves allow to establish the etiology of the condition, making further investigations necessary. When LETM secondary to CHIKV infection is suspected, confirmation requires a CHIKV IgM antibody titer in CSF or RT-PCR (12, 13). Positivity in either of these tests will confirm the neuroinvasive form of the condition associated with the typical clinical form. In addition, the presence of antibodies to Zika virus and Dengue virus should be ruled out. The latter is known to cross-react with CHIKV IgM antibodies, especially in endemic regions (12–16). Barreto et al. also stress the need to collect serum titers of anti-aquaporin 4, anti-P-ribosomal and antiphospholipid antibodies due to their association with neuropsychiatric disorders secondary to SLE (2, 3, 14).

As described in the literature, a variety of patterns may be observed when neuroimaging CHIKV-infected patients, depending on the neurological syndrome. However, when exploring the myelopathy spectrum, Silva et al. and Ganesan et al. found T2 hyperintensity and restriction of contrast diffusion and enhancement, with a preference for the periventricular white matter (6, 17). Others have described extensive T2 hyperintensity along the spinal cord, compatible with LETM (18), and enhanced intramedullary lesions (19), matching the neuraxis MRI findings of our case and supporting the diagnosis of CHIKV infection. Interestingly, Nobrega et al. (20) described a 69-year-old female patient with Chikungunya myelopathy and “glass eel” pattern on MRI (a longitudinally extensive hypersignal on the peripheral zone of the spinal cord on sagittal T2 images sparing the central gray matter).

The suspicion of neurological involvement secondary to CHIKV infection was reinforced in our case by the presence of pleocytosis, hypoglycorrhachia and proteinorachia, positivity for CHIKV IgM antibodies in CSF, and negativity for Zika and Dengue antibodies. It should be noted that we used molecular techniques to identify possible cross-reactivity, which, though rare, has been reported for other arboviruses (21). The absence of anti-aquaporin 4 and antiphospholipid antibodies contrasts with the findings of other authors who mostly associated SLE-related LETM with optic neuromyelitis or anti-aquaporin 4 antibody positivity (2, 4, 22, 23).

To our knowledge, neurochikungunya in SLE has not been thoroughly investigated before, making this a highly relevant report. Though rare, the combination of these two conditions poses a considerable potential for morbidity and can seriously compromise functionality.

Our case illustrates the complex interaction between SLE and neuroinvasive CHIKV infection, resulting in severe LETM. The late neurological presentation (14 years after the diagnosis of SLE), the positivity for CHIKV IgM antibodies and the imaging findings show the relevance of performing thorough diagnostic investigations in cases coming from endemic regions like Brazil and other Latin American countries.

The optimal treatment remains unclear. Our patient presented motor sequelae predominantly in the lower limbs despite aggressive immunomodulatory therapy with plasma exchange, corticosteroid pulse and cyclophosphamide, suggesting CHIKV may be associated with high levels of inflammatory cytokines (interleukin-1b, interferon type I, interleukin-6, tumor necrosis factor-α), potentially enhancing the immune-mediated process in SLE. Histological evidence of CHIKV neurotropism is an ambiguous finding, but CSF positivity has been demonstrated in other cases (17, 24). Post-infectious neuropathy develops from autoimmune reactions to the infectious agent, which cross-react with neural antigens of Schwann cells/myelin or axons in the peripheral nerve (17). Such autoantigen-like viral epitopes can induce antibody production, amplifying the already existing immune response in SLE. The asymptomatic period from acute illness to the development of myeloradiculoneuropathy suggests an immune-mediated condition secondary to infection, rather than direct invasion (19).

## Conclusion

The present case contributes to current knowledge of the array of neurological manifestations observed in SLE patients and draws the attention of physicians in endemic regions like Brazil to the need for including CHIKV infection, though rare, in the differential diagnosis. It also emphasizes the high rates of functional disability, morbidity and mortality associated with inflammatory myelopathy. Early and accurate diagnosis, targeted treatment, and intensive rehabilitation can minimize the impact of this threatening condition.

## Data Availability

The datasets presented in this article are not readily available because of ethical and privacy restrictions. Requests to access the datasets should be directed to the corresponding author.
